# Supplementary material for: Feasibility, efficacy, and perceptions of an online writing intervention in patients with depressive disorders: A randomized, multi-methods pilot study
Source: PLOS Ment Health. 2025 Jul 31;2(7):e0000245. doi: 10.1371/journal.pmen.0000245 (PMC12798339; doi:10.1371/journal.pmen.0000245)
Supplement: S1 File — (DOCX) [file pmen.0000245.s001.docx]

# S1 File: Study Protocols and Checklists

***Study Protocol***

**Online Writing Intervention for Major Depressive Disorder**
**ClinicalTrials.gov ID:** NCT06699719
**Sponsor:** Centre for Addiction and Mental Health
**Information provided by:** Centre for Addiction and Mental Health
(Responsible Party)

**Last Update Posted:** 2024-11-21

**Study Overview

Brief Summary**
The goal of this trial is to examine the feasibility of administering an online writing intervention to patients diagnosed with Major Depressive Disorder, and whether this intervention impacts symptoms of depression. It also examines the impact of the intervention on symptoms of anxiety, personal functioning, and perceived problem complexity, and how participants experience the intervention. The main questions the trial aims to answer are:

- Will participants randomized to the online writing intervention complete at least three sessions?
- Is the writing intervention associated with a reduction in symptoms of depression and anxiety, perceived problem complexity, and/or an improvement in personal functioning?
- What are participants' perceptions of the online writing intervention, including both positive and negative experiences?

Participants will:

- Complete four sessions of an online writing intervention or no writing intervention over the course of one week
- Complete questionnaires before and after the intervention, and at a one-month follow-up
- Complete qualitative interviews probing into their experiences with the intervention

**Detailed Description**

*I. Introduction and Background*

In a 2012 Statistics Canada Survey, 1.6 million Canadians reported that their needs for mental health services were unmet. In Ontario, almost 60% of mental health concerns are related to Major Depressive Disorder (MDD), representing an estimated excess of 210 million dollars in healthcare costs. Psychotherapy is an effective and enduring option for treating depression, but in Canada, its availability is limited. Increasing the number of psychotherapists does not seem to be an adequate solution to this issue; despite an increase in psychotherapy provision in the past decade, many Canadians are still in need of psychotherapy.

One solution is to develop innovative therapies that can be delivered reliably at low costs to large populations, for example, using the internet. Expressive writing (EW) is a simple intervention involving a series of journaling tasks where individuals write their deepest thoughts and feelings about a negative issue or problem. There is a large literature examining the various physical and psychological benefits associated with EW among different populations. EW appears to be a promising intervention for MDD. In one study of clinically depressed patients, EW completed in person for 20 minutes over three consecutive days resulted in less depressive symptoms relative to control writing, and this effect persisted after one month. Importantly, EW does not require manuals or highly trained clinicians, making it cost-effective and amenable to being administered remotely. Preliminary studies of its online administration with depressed individuals have produced encouraging findings.

*II. Rationale and Study Objectives*

This study determines whether it is feasible to administer EW online to patients with MDD, and whether patients find it useful.

Feasibility concerns. Although the open-ended nature of EW makes it easy for patients to complete remotely (i.e., without the guidance or presence of a clinician), it presents a risk of drop-out particularly in severely depressed patients, due to a lack of motivation and other factors. The investigators assess the feasibility of administering EW remotely (i.e., online or by mail) to participants seeking treatment at CAMH for Major Depression. They also examine participants' perceptions of EW (i.e., its potential benefits and limitations), as well as their motivation and engagement with the intervention.

Efficacy concerns in a target patient population. In various studies, EW has reduced symptoms of depression. However, other studies have reported no benefits of EW or even adverse effects for individuals with severe levels of depression. These inconsistent findings have prompted researchers to caution against using EW in severely depressed samples. They suggest that writing facilitates depressive rumination (i.e., persistent and recursive thinking about the depressive episode), leading to a worsening of symptoms. Thus, it is also possible that unstructured treatments like EW are not helpful for individuals with depression, particularly if administered online. For this reason, the investigators also assess the efficacy of online EW in a sample of CAMH patients who are diagnosed with depression. Specifically, they examine its impact on symptoms of depression and anxiety, personal functioning, and perceived complexity of personal problems.

*III. Aims and Hypotheses*

The investigators conduct a multi-methods study to assess the feasibility and efficacy of administering EW online to patients with MDD. They also examine perceptions of its impact on emotional and cognitive wellbeing. To achieve these aims, the investigators randomize patients into one of two groups: one group is prompted to complete four sessions of EW online, whereas the other group is not asked to complete this task. Although control conditions in studies of EW often involve sessions of writing about non-emotional topics, the investigators do not administer a control writing task due to their focus on feasibility. The aim is to examine whether a group of patients with MDD would complete a writing intervention online, which requires a comparison of their study completion rates and outcomes to a group not asked to complete any writing at all. Nevertheless, both groups complete self-reported measures of outcomes. The primary outcome related to efficacy is reduction in MDD symptoms; secondary outcomes include symptoms of Generalized Anxiety Disorder (GAD), functional impairment, and the perceived complexity of personal problems. Participants complete these measures before and after the EW task, and at a one-month follow up. The investigators examine the rate of EW completion and adherence to the suggested writing duration, as well as its impact on outcomes over time. They also conduct qualitative interviews which ask EW participants about their experiences with the intervention, with a focus on perceived benefits and limitations, and its emotional and cognitive effects.

The investigators propose that online administration is feasible if 70% of participants randomized to the intervention complete at least three 20-minute sessions of writing. EW is useful if there is a significant reduction in MDD symptoms over time, as compared to the control group, which either emerges after the intervention or at the one-month follow-up. Based on research finding temporary increases in emotional distress related to EW, it is also possible EW participants might report increased symptoms immediately following the intervention. However, changes in functional impairment or perceived problem complexity could take some time to emerge, so changes related to these outcomes are expected to emerge at the one month follow-up. In qualitative interviews, participants are expected to report EW to be beneficial, particularly as related to its emotional or cognitive effects.

*IV. Methods*

Participants The investigators recruit outpatients from the Centre for Addiction and Mental Health. Patients are referred to the study by psychiatrists during initial or follow-up appointments, or by psychotherapists either before the start or after the end of group psychotherapy. Inclusion criteria are adults aged 18 or older, who were assessed by a psychiatrist and received a diagnosis of MDD with or without another co-morbid condition. Participants also require access to the internet and an email address at which they could receive study materials. Patients are excluded if they cannot understand, speak, or write in English. Clinicians are also asked not to refer patients into the study if they had cognitive impairments that could prevent them from being able to complete a writing task. Clinicians are also asked not to refer patients reporting suicidal thoughts and behaviours or other mental health symptoms which could put them at risk for negative outcomes when completing the intervention remotely. Patients are not excluded if they are receiving treatment for MDD (e.g., antidepressants, group CBT).

*Measures Demographics:* Participants self-report their demographic characteristics, which included their age, gender, racial or ethnic background, and postal code, which was used to derive socioeconomic status.

*Patient Health Questionnaire (PHQ-9):* The PHQ-9 is a self-reported measure of MDD symptom severity; it measures the frequency of 9 symptoms on a scale from 0 (not at all) to 3 (nearly everyday). Scores are summed to reflect MDD symptom severity, and they range from 0-27, with higher scores indicating greater severity.

*Generalized Anxiety Disorder (GAD-7):* The GAD-7 is a self-reported measure of GAD symptom severity; it measures the frequency of 7 symptoms on a scale from 0 (not at all) to 3 (nearly everyday). Summed scores range from 0-21, with higher scores indicating greater severity.

*WHO-Disability Assessment Schedule (WHODAS):* WHODAS is a self-reported measure of functional impairment. It measures the degree of difficulty from the past month related to 12 activities in domains of cognition, mobility, self-care, and social participation. Activities are measured on a scale from 0 (none) to 4 (extreme or cannot do), and scores are summed to reflect overall functional impairment. They range from 0-48, with higher scores indicating greater impairment.

*Problem Complexity Questionnaire (PCQ):* PCQ measures the perceived complexity of personal problems. It consists of 8 statements reflecting the complexity of problems, and participants rate each item on a scale from 1 (completely disagree) to 4 (completely agree). Scores range from 8 to 32, with higher scores indicating higher perceptions of problem complexity.

**Procedures**

The study is approved by CAMH's Research Ethics Board. Patients referred to the study are provided with a high-level description of study aims (i.e., "evaluating the extent to which completing questionnaires or a writing task is useful for people with depression"). Prior to enrolment, all participants are given information about study procedures and risks, and they provide their written informed consent. Once enrolled, participants are allocated to EW or control with 4-block randomization and they are sent the first study questionnaire consisting of the demographics form, PHQ-9, GAD-7, WHODAS, and PCQ. Their electronic health records are also accessed to extract information about their diagnoses and current treatment. After completing the first questionnaire, participants assigned to EW are sent instructions to complete the first EW session on the next day. Instructions are accompanied by a text box, in which participants can write freely. Although participants are instructed to write for 20 minutes, they are also told during that they could pause or stop the writing task at any time (e.g., if they felt unwell). EW participants are provided with the same instructions on the next three days. Participants who are allocated to control are not provided with the EW instructions. After one week (for the control group) or on the next day that participants complete their fourth EW session (for the EW group), participants receive a second questionnaire containing the PHQ-9, GAD-7, WHODAS, and PCQ. One month after completing the second questionnaire, they receive a third questionnaire containing the same measures. All study materials (i.e., questionnaires, EW sessions) are administered via RedCap, a web-based application for data collection available at CAMH, and study instructions are sent to participants by email. If participants do not complete an EW session or follow-up questionnaire within three days of receiving it (or after one week for the first questionnaire), participants who agree to receive reminders by text message or email are sent up to two reminders. Participants who do not respond or complete the relevant study components after two reminders are assumed to have dropped out of the study and are no longer contacted. However, participants who respond to reminders or complete the questionnaires or EW sessions within any period of time are included.

After completing the third questionnaire, all EW participants are invited to participate in a brief qualitative interview. Qualitative interviewing is guided by the question of how individuals with MDD experience online EW, barriers to its completion, perceived benefits and limitations, emotional and cognitive effects, and suggestions for improvement. At the end of the study, control participants are also given an opportunity to complete the EW task, if they wish.

**Statistical Analysis**

The investigators examine the baseline demographic and clinical characteristics of the sample and compare them between the control and EW groups. Differences in categorical variables are compared with Chi-squared tests. Due to small samples in some racial or ethnic categories, this variable is recoded into a binary representation when testing for differences between groups. Continuous variables that are normally distributed are compared with t-tests, whereas Wilcoxon-signed rank tests are used for continuous variables with non-parametric distributions.

The investigators examine how many EW participants completed at least three sessions, how many wrote for at least 20 minutes, the median length of time spent writing, the median word count of EW tasks, and average time intervals between writing sessions. Intention-to-treat analysis involves generating mixed effect models with maximum likelihood estimation using all participants in the EW group and all participants in the control group who complete the first (baseline) questionnaire. The investigators generate four models to examine the impact of condition, time, and a condition by time interaction on each outcome (i.e., PHQ-9, GAD-7, WHODAS, PCQ). Time is treated as a categorical factor, to account for the possibility of non-linear effects. For any models with evidence of this interaction, estimated means are plotted to inspect trajectories, with 95% confidence intervals to gauge effects. In a per-protocol analysis, the investigators generate the same mixed effect models adding number of completed sessions as a predictor, to determine whether completing fewer sessions impacts outcomes (setting all control participants to 0).

The approach to analysing qualitative interviews is guided by reflexive thematic analysis (Braun & Clark, 2019), which is a recursive and constructive process of identifying and analysing themes. Given the focus on emotional and cognitive effects, the investigators adopt a predominantly theoretical or deductive approach, to provide a nuanced account of how participants experienced these effects of EW. To accommodate the diversity of experiences, the investigators incorporated inductive analysis. Analysis is primarily semantic and essentialist, since the investigators wish to understand participants' explicit experiences or accounts of EW, rather than deriving latent themes.

**Conditions:** Major Depression, Generalized Anxiety Disorder (GAD) **Intervention:** Behavioural – Expressive Writing **Other Study ID Numbers:** 100/2018 **Study Start (Enrolment - Actual):** 2019-03-27 **Primary Completion (Actual):** 2022-06-14 **Enrollment (Actual):** 63 **Study Type:** Interventional **Phase:** NA **Contacts and Locations**This study has 1 location
Canada – Toronto, Ontario, M5T 1R8
Centre for Addiction and Mental Health

**Eligibility Criteria**

*Description*

Ages Eligible for Study: 18 Years and older
Sexes Eligible for Study: All
Accepts Healthy Volunteers: No

**Design Details**

Primary Purpose: Treatment
Allocation: Randomized
Interventional Model: Single Group Assignment
Masking: Single
Interventional Model Description: Only one intervention arm is available to study participants (i.e., an online EW intervention), and participants are randomized to one of two arms: 1) the intervention, which is to complete four sessions of writing online or 2) no intervention (i.e., control).

**Participant Arms**

No Intervention:
Control. Participants are not asked to complete the online writing intervention

Experimental:
Expressive writing. Participants are asked to complete four 20-minute sessions of an online writing task on consecutive days
Behavioral: Participants are asked to complete four 20-minute sessions of a writing task on consecutive days, which is sent to their email address via an online link. Participants are instructed "for the next 20 minutes to please use the provided box to write their very deepest thoughts and feelings about a negative issue or personal problem that they are currently being affected by. In their writing, they are encouraged to let go and explore their emotions and thoughts regarding this issue or problem, and to not worry about spelling or grammar. They are asked to consider setting a timer or have a clock handy to make sure they write for 20 minutes." Instructions are accompanied by a text box, which allows participants to write freely for the allotted time.

Other Names: EW; online writing intervention

**What is the study measuring?***Primary outcome measures*

- Feasibility of completing EW: Feasibility is defined as 70% of EW participants completing at least three 20-minute sessions of EW. Time frame: Feasibility is determined immediately following the completion of the third writing session, which is at minimum after 3 days or one week, on average.
- Depressive symptoms: Severity of depressive symptoms, as assessed via the Patient Health Questionnaire (PHQ-9); values range from 0-27 with higher scores indicating greater symptom severity (or worse outcome). Time frame: On the next day after the fourth online writing session for participants assigned to EW or after one week for control participants, and at a one-month

*Secondary outcome measures*

- Anxiety symptoms: Severity of anxiety symptoms, as assessed via the Generalized Anxiety Disorder (GAD-7) questionnaire; scores range from 0-21with higher scores indicating greater symptom severity (or worse outcomes). Time frame: On the next day after the fourth online writing session for participants assigned to EW or after one week for control participants, and at a one-month
- Impaired functioning: Functional impairment as assessed by the12 item WHO-Disability Assessment Schedule (WHODAS); scores range from 0-48 with higher scores indicating greater impairment (or worse outcome). Time frame: On the next day after the fourth online writing session for participants assigned to EW or after one week for control participants, and at a one-month
- Problem Complexity: Perceived complexity of personal problems as assessed by the Problem Complexity Questionnaire (PCQ); scores range from 0-36 with higher scores indicating greater perceived problem complexity (or worse outcome). Time frame: On the next day after the fourth online writing session for participants assigned to EW or after one week for control participants, and at a one-month

**Collaborators and Investigators**

Centre for Addiction and Mental Health
Principal Investigator: Benoit H Mulsant, MD, MS, FRCPC, DLFAPA, Centre for Addiction and Mental Health

**Publications**
These publications are provided voluntarily by the person who enters information about the study and may be about anything related to the study.

Gratzer D, Goldbloom D. Making Evidence-Based Psychotherapy More Accessible in Canada. Can J Psychiatry. 2016 Oct;61(10):618-23. doi:10.1177/0706743716642416. Epub 2016 Apr 5. No abstract available.

Hayes AM, Feldman GC, Beevers CG, Laurenceau JP, Cardaciotto L, Lewis-SmithJ. Discontinuities and cognitive changes in an exposure-based cognitive therapy for depression. J Consult Clin Psychol. 2007 Jun;75(3):409-421. doi:10.1037/0022-006X.75.3.409.

Sbarra DA, Boals A, Mason AE, Larson GM, Mehl MR. Expressive Writing Can Impede Emotional Recovery Following Marital Separation. Clin Psychol Sci.2013 Mar 18;1(2):120-134. doi: 10.1177/2167702612469801.

Kovac SH, Range LM. Does writing about suicidal thoughts and feelings reduce them? Suicide Life Threat Behav. 2002 Winter;32(4):428-40. doi:10.1521/suli.32.4.428.22335.

Sloan DM, Marx BP, Epstein EM, Lexington JM. Does altering the writing instructions influence outcome associated with written disclosure? Behav Ther. 2007 Jun;38(2):155-68. doi: 10.1016/j.beth.2006.06.005. Epub 2007 Jan18.

Gortner EM, Rude SS, Pennebaker JW. Benefits of expressive writing in lowering rumination and depressive symptoms. Behav Ther. 2006Sep;37(3):292-303. doi: 10.1016/j.beth.2006.01.004. Epub 2006 May 30.

Krpan KM, Kross E, Berman MG, Deldin PJ, Askren MK, Jonides J. An everyday activity as a treatment for depression: the benefits of expressive writing for people diagnosed with major depressive disorder. J Affect Disord. 2013 Sep 25;150(3):1148-51. doi: 10.1016/j.jad.2013.05.065. Epub 2013 Jun 18.

Frattaroli J. Experimental disclosure and its moderators: a meta-analysis. Psychol Bull. 2006 Nov;132(6):823-65. doi: 10.1037/0033-2909.132.6.823.

Chiu M, Lebenbaum M, Cheng J, de Oliveira C, Kurdyak P. The direct healthcare costs associated with psychological distress and major depression: A population-based cohort study in Ontario, Canada. PLoS One. 2017 Sep 5;12(9):e0184268. doi: 10.1371/journal.pone.0184268. eCollection 2017.

Baikie KA, Geerligs L, Wilhelm K. Expressive writing and positive writing for participants with mood disorders: an online randomized controlled trial. J Affect Disord. 2012 Feb;136(3):310-9. doi: 10.1016/j.jad.2011.11.032. Epub 2011 Dec 30.

**Terms Related to this Study**

Keywords Provided by Centre for Addiction and Mental Health:

- Expressive writing
- Major Depression
- Online intervention
- Digital mental health

Additional Relevant MeSH Terms:

- Behavioral Symptoms
- Mood Disorders
- Mental Disorders
- Depression
- Depressive Disorder
- Depressive Disorder, Major
- Anxiety Disorders

***Suicide Risk Management Protocol***

**Online Writing Intervention for Major Depressive Disorder**

Any clients who respond with a 1 or higher on the PHQ-9 suicide item during their online assessment will be prompted to complete Questions #1-2, Part A (see below). If the summed score to these two questions is less than or equal to 1, participants will continue with the assessment, and no further action will be taken. If the summed score to these two questions is greater than 1, participants will be prompted to complete Questions #3-5, Part B (see below). If a participant’s summed score for Part B is less than 3, participants will continue with the assessment, and no further action will be taken. If a participant’s summed score for Part B is greater than or equal to 3, within one day, study staff will notify the participant’s referring psychiatrist/clinician, so that the psychiatrist/clinician can follow up with the participant (i.e., complete a risk assessment and deploy help or resources, if necessary).

If the referring psychiatrist/clinician does not respond or indicates that they no longer have a professional relationship with the participant, study staff will inform the study Principal Investigator. Study staff will contact the participant to carry out a risk management protocol. This protocol begins with a risk factor assessment (i.e., assessing suicide history, impulsiveness, alcohol use, social support and other potential risk factors). If many risk factors are identified, study staff proceeds to collect information on characteristics of suicidal ideation (frequency, persistence, duration, motivation, deterrents, steps taken), and characteristics of contemplated attempts to identify the risk of self harm or suicide as low, moderate, high or immediate.

If the risk is immediate, study staff will keep the participant on the study phone and call 911 from a personal phone. If the risk is high or moderate, study staff will contract for safety with the participant, and schedule to call back within 1 or 2 hours respectively. In the meantime, staff will call the Principal Investigator’s cellphone to notify them of the situation and for further guidance (i.e., to call the participant back at the scheduled time). If the study Principal Investigator is unavailable, study staff will contact another study co-investigator/psychiatrist. If the co-investigator is unavailable, study staff will contact CAMH’s Emergency Department (ED), to notify an ED psychiatrist of the situation and discuss next steps.

PART A
Earlier, you mentioned some thoughts about death. The following questions will ask about your attitude towards living and dying.

1. Can you tell me about your wish to live today? Is it moderate to strong? Weak? Or None?
0 Moderate to strong
1 Weak
2 None

2. Can you tell me about your wish to die? Is it moderate to strong? Weak? Or none?
0 Moderate to strong
1 Weak
2 None

PART B
3. Would you say today that your reasons for living outweigh your reasons for dying?
0 For living outweighing for dying
1 About equal
2 For dying outweigh for living

4. What is your current desire to to actively harm or kill yourself?
0 None
1 Weak
2 Moderate to strong

5. Today, would you take precautions necessary to save your life? Would you take medicine to save your life? Would you drive safely to keep yourself alive?
0 Would take precautions to save life
1 Would leave life/death to chance (e.g., carelessly crossing a busy street)
2 Would avoid steps necessary to safe or maintain life (e.g., diabetic ceasing to take insulin)

**CONSORT 2010 checklist of information to include when reporting a randomised trial***

| **Section/Topic** | **Item No** | **Checklist item** | **Page No** |
| --- | --- | --- | --- |
| **Title and abstract** | | | |
|  | 1a | Identification as a randomised trial in the title | 1 - title |
|  | 1b | Structured summary of trial design, methods, results, and conclusions (for specific guidance see CONSORT for abstracts) | 2-3 |
| **Introduction** | | | |
| Background and objectives | 2a | Scientific background and explanation of rationale | 3-8 |
|  | 2b | Specific objectives or hypotheses | 8-9 |
| **Methods** | | | |
| Trial design | 3a | Description of trial design (such as parallel, factorial) including allocation ratio | 12 |
|  | 3b | Important changes to methods after trial commencement (such as eligibility criteria), with reasons | NA |
| Participants | 4a | Eligibility criteria for participants | 9-10 |
|  | 4b | Settings and locations where the data were collected | 9-10 |
| Interventions | 5 | The interventions for each group with sufficient details to allow replication, including how and when they were actually administered | 12-13 |
| Outcomes | 6a | Completely defined pre-specified primary and secondary outcome measures, including how and when they were assessed | 10-11 |
|  | 6b | Any changes to trial outcomes after the trial commenced, with reasons | NA |
| Sample size | 7a | How sample size was determined | 20 |
|  | 7b | When applicable, explanation of any interim analyses and stopping guidelines | NA |
| Randomisation: |  |  |  |
| Sequence generation | 8a | Method used to generate the random allocation sequence | 12 |
|  | 8b | Type of randomisation; details of any restriction (such as blocking and block size) | 12 |
| Allocation concealment mechanism | 9 | Mechanism used to implement the random allocation sequence (such as sequentially numbered containers), describing any steps taken to conceal the sequence until interventions were assigned | NA |
| Implementation | 10 | Who generated the random allocation sequence, who enrolled participants, and who assigned participants to interventions | 13 |
| Blinding | 11a | If done, who was blinded after assignment to interventions (for example, participants, care providers, those assessing outcomes) and how | NA |
|  | 11b | If relevant, description of the similarity of interventions | NA |
| Statistical methods | 12a | Statistical methods used to compare groups for primary and secondary outcomes | 14-15 |
|  | 12b | Methods for additional analyses, such as subgroup analyses and adjusted analyses | 15 |
| **Results** | | | |
| Participant flow (a diagram is strongly recommended) | 13a | For each group, the numbers of participants who were randomly assigned, received intended treatment, and were analysed for the primary outcome | 16 |
|  | 13b | For each group, losses and exclusions after randomisation, together with reasons | 17 |
| Recruitment | 14a | Dates defining the periods of recruitment and follow-up | 9 |
|  | 14b | Why the trial ended or was stopped | NA |
| Baseline data | 15 | A table showing baseline demographic and clinical characteristics for each group | 18 |
| Numbers analysed | 16 | For each group, number of participants (denominator) included in each analysis and whether the analysis was by original assigned groups | 17-18 |
| Outcomes and estimation | 17a | For each primary and secondary outcome, results for each group, and the estimated effect size and its precision (such as 95% confidence interval) | 19-20 |
|  | 17b | For binary outcomes, presentation of both absolute and relative effect sizes is recommended | NA |
| Ancillary analyses | 18 | Results of any other analyses performed, including subgroup analyses and adjusted analyses, distinguishing pre-specified from exploratory | 21-25 |
| Harms | 19 | All important harms or unintended effects in each group (for specific guidance see CONSORT for harms) | NA |
| **Discussion** | | | |
| Limitations | 20 | Trial limitations, addressing sources of potential bias, imprecision, and, if relevant, multiplicity of analyses | 30-31 |
| Generalisability | 21 | Generalisability (external validity, applicability) of the trial findings | NA |
| Interpretation | 22 | Interpretation consistent with results, balancing benefits and harms, and considering other relevant evidence | 25-30 |
| **Other information** | | |  |
| Registration | 23 | Registration number and name of trial registry | 3, 12 |
| Protocol | 24 | Where the full trial protocol can be accessed, if available | 3, 12 |
| Funding | 25 | Sources of funding and other support (such as supply of drugs), role of funders | NA |

Citation: Schulz KF, Altman DG, Moher D, for the CONSORT Group. CONSORT 2010 Statement: updated guidelines for reporting parallel group randomised trials. BMC Medicine. 2010;8:18.
© 2010 Schulz et al. This is an Open Access article distributed under the terms of the Creative Commons Attribution License (<http://creativecommons.org/licenses/by/2.0>), which permits unrestricted use, distribution, and reproduction in any medium, provided the original work is properly cited.
