# Supplementary material for: Feasibility, efficacy, and perceptions of an online writing intervention in patients with depressive disorders: A randomized, multi-methods pilot study
Source: PLOS Ment Health. 2025 Jul 31;2(7):e0000245. doi: 10.1371/journal.pmen.0000245 (PMC12798339; doi:10.1371/journal.pmen.0000245)
Supplement: S2 File — (DOCX) [file pmen.0000245.s002.docx]

# S2 File

***Expressive writing interview guide***

Completion: Were you able to complete any of the writing sessions?
Prompts: How many did you complete? Can you describe any reasons for not completing all the writing sessions? Is there a particular aspect of the writing task that made you decide not to complete it?

*(If participants completed at least 1 session, proceed to next questions)*

Remembering: Did you find it difficult to remember to complete the writing sessions each day?
Prompts: Did you receive any text/email reminders? Did you find them helpful, i.e., did you complete additional writing sessions as a result of these reminders? If so, how many?

Accessibility: Did you find this writing task accessible to you? Were there any barriers to you completing it?

Clarity: How did you find the writing task instructions?
Prompts: Were instructions clear? Why or why not? Do you think you would have benefitted from more specific instructions or guides on things to write each day? Did you like having the freedom to write anything you wanted?

Ease: Did you find it easy to express your emotions and thoughts during writing? Why or why not?

Honesty: Were you able to be open or candid when writing? Why or why not?

Motivation: How motivated were you to complete the writing task?
Prompts: Were you dragging your feet beforehand, did you have to make yourself do it, or was it easy to get going? Were you dreading having to do the writing task or did you look forward to it?

Feelings during: While you were writing, how did you feel?
Prompts: Did you find it pleasant or did it make you feel positively? Did this encourage you to write more or continue? Did you find that the writing task was unpleasant or did it make you feel negatively? Did this prevent you from completing the writing task (made you stop/finish early/not complete subsequent sessions)?

Comfort: Did you feel comfortable while writing? Why or why not?
Prompts: Did you feel awkward? Were you concerned about privacy? Did you feel comfortable sharing it with us? Would you have preferred to keep your writing just for yourself?

Feelings after: How did you feel after the writing task sessions?
Prompt: Did the way you felt encourage you to/prevent you from participating in further writing sessions?

Usefulness: Did you find the writing task useful or helpful? Why or why not?
Prompts: Did you notice or consider emotions or thoughts that you had not considered before? Did you feel like you learned something new about yourself, your life, or someone else? Did you feel overwhelmed by what you were writing about? Did writing make you more confused about things in your life?

Engagement: How engaged were you during writing?
Prompts: Did you find it easy to keep your attention focused on the task? Did you feel the time went by quickly? Did you feel like your mind was wandering? Did you feel like you were just waiting for the time to be up? Did you find that you had nothing to write about?

Sessions: How did you find the timing and spacing of writing sessions?
Prompts: Would you have preferred not to do it everyday and every few days? Would you prefer for it to be longer or shorter? Did you continue writing on your own once the sessions were completed?

General:
Did you find any aspects of the writing task positive, useful, or enjoyable? Which ones? Why?
Did you find any aspects of the writing task negative, unhelpful, or unpleasant? Which ones? Why?
Is there anything else about your experience with the writing task that you’d like to share?
