# Supplementary material for: Feasibility, efficacy, and perceptions of an online writing intervention in patients with depressive disorders: A randomized, multi-methods pilot study
Source: PLOS Ment Health. 2025 Jul 31;2(7):e0000245. doi: 10.1371/journal.pmen.0000245 (PMC12798339; doi:10.1371/journal.pmen.0000245)
Supplement: S3 File — (DOCX) [file pmen.0000245.s003.docx]

# S3 File

## *Clinical characteristics extracted from health records*

| ***Variable^a^*** | ***N (%)*** |
| --- | --- |
| Diagnoses^b^ |  |
| Major Depressive Disorder (MDD) | 41 (77%) |
| Persistent depressive disorder (PDD)/Dysthymia | 12 (22%) |
| Generalized Anxiety Disorder (GAD) | 20 (41%) |
| Social anxiety disorder/Social phobia | 10 (19%) |
| Panic disorder | 2 (4%) |
| PTSD/Trauma or stressor-related disorder | 3 (6%) |
| Other disorder^c^ | 11 (21%) |
| Psychotropic medication use | 28 (53%) |
| Psychotherapy |  |
| None | 30 (57%) |
| Prior to the study | 15 (28%) |
| During the study | 6 (11%) |

^a^One patient did not consent to the review of their health records.

^b^Most patients had multiple diagnoses recorded in their health records; all patients included in analyses had a diagnosis of MDD or PDD, including one patient whose health record indicated a diagnosis of “depression” (PHQ-9 score = 24) (categorized here as ‘Other’). One patient with GAD did not have a Depressive Disorder documented in their health record; this patient was excluded from analyses and is not represented here.
^c^Other disorder additionally includes cannabis use disorder, opioid use disorder, illness anxiety disorder, sleep disorder, adjustment disorder with depressed mood, and borderline personality traits
