# Supplementary material for: Feasibility, efficacy, and perceptions of an online writing intervention in patients with depressive disorders: A randomized, multi-methods pilot study
Source: PLOS Ment Health. 2025 Jul 31;2(7):e0000245. doi: 10.1371/journal.pmen.0000245 (PMC12798339; doi:10.1371/journal.pmen.0000245)
Supplement: S4 File — (DOCX) [file pmen.0000245.s004.docx]

# S4 File

***Post-hoc power analyses***To facilitate an interpretation of our findings related to EW efficacy, we completed a *post-hoc* power analysis based on a two-factor repeated measures design, *n*=23 in each group, 80% power, *p*=0.05, and assuming sphericity. Based on the statistic *f* of effect size for repeated-measures ANOVA (Cohen, 1988), our analyses were powered to detect large effects of EW on the condition by time interaction (*f*=0.47) at any follow-up (either immediately after writing or at the one-month follow-up). Power analysis was completed in R using the WebPower package (Zhang et al., 2018; Zhang & Yuan, 2018).

**References**
Cohen, J. (1988). *Statistical power analysis for the behavioral sciences* (2nd Ed). Hillsdale, NJ: Lawrence Erlbaum Associates.

Zhang, Z., Mai, Y., Yang, M., & Zhang, M. Z. (2018). Package ‘WebPower’. *Basic and advanced statistical power analysis version, 72*, 555.

Zhang, Z., & Yuan, K.-H. (2018). *Practical Statistical Power Analysis Using Webpower and R* (Eds). Granger, IN: ISDSA Press.
