# Supplementary material for: Feasibility, efficacy, and perceptions of an online writing intervention in patients with depressive disorders: A randomized, multi-methods pilot study
Source: PLOS Ment Health. 2025 Jul 31;2(7):e0000245. doi: 10.1371/journal.pmen.0000245 (PMC12798339; doi:10.1371/journal.pmen.0000245)
Supplement: S5 File — (DOCX) [file pmen.0000245.s005.docx]

**S5 File**

***Per-protocol analyses: Results***

Results for per-protocol analyses are provided in Table A. Accounting for number of completed sessions and overall EW text lengths do not qualitatively change findings from the intention-to-treat analysis, despite the emergence of some main effects of session number and condition. For the effects of EW on PHQ-9 scores, increased sessions were associated with more severe symptoms (*r*=1.76, *SE*=0.84, *p*=.042).

**Table A.** Results for per-protocol analyses, accounting for session number and EW text length

| ***Outcome*** | ***Session number*** | ***Condition*** | ***Time*** | ***Interaction*** |
| --- | --- | --- | --- | --- |
| PHQ-9 | *F*=4.35, *p*=.042 | *F*=6.81, *p*=.011 | *F*=2.480, *p*=.090 | *F*=0.04, *p*=.963 |
| GAD-7 | *F*=2.13, *p*=.150 | *F*=3.42, *p*=.069 | *F*=2.80, *p*=.067 | *F*=0.24, *p*=.787 |
| WHODAS | *F*=3.04, *p*=.087 | *F*=5.08, *p*=.028 | *F*=1.27, *p*=.286 | *F*=1.15, *p*=.320 |
| PCQ | *F*=13.19, *p*=.001 | *F*=10.21, *p*=.002 | *F*=0.70, *p*=.502 | *F*=0.53, *p*=.588 |
| ***Outcome*** | ***Text length*** | ***Condition*** | ***Time*** | ***Interaction*** |
| PHQ-9 | *F*=0.20, *p*=.656 | *F*=0.51, *p*=.476 | *F*=2.012, *p*=.140 | *F*=0.12, *p*=.890 |
| GAD-7 | *F*=0.00, *p*=.999 | *F*=0.61, *p*=.438 | *F*=2.62, *p*=.079 | *F*=0.17, *p*=.848 |
| WHODAS | *F*=0.02, *p*=.963 | *F*=0.90, *p*=.346 | *F*=1.35, *p*=.264 | *F*=1.02, *p*=.365 |
| PCQ | *F*=2.14, *p*=.150 | *F*=1.10, *p*=.299 | *F*=0.73, *p*=.484 | *F*=0.80, *p*=.454 |

After controlling for session number, the effect of condition was statistically significant at each time point. Inspecting a plot of estimated marginal means (provided below), it appears that the EW group reported less severe MDD symptoms than the control group, including at baseline.


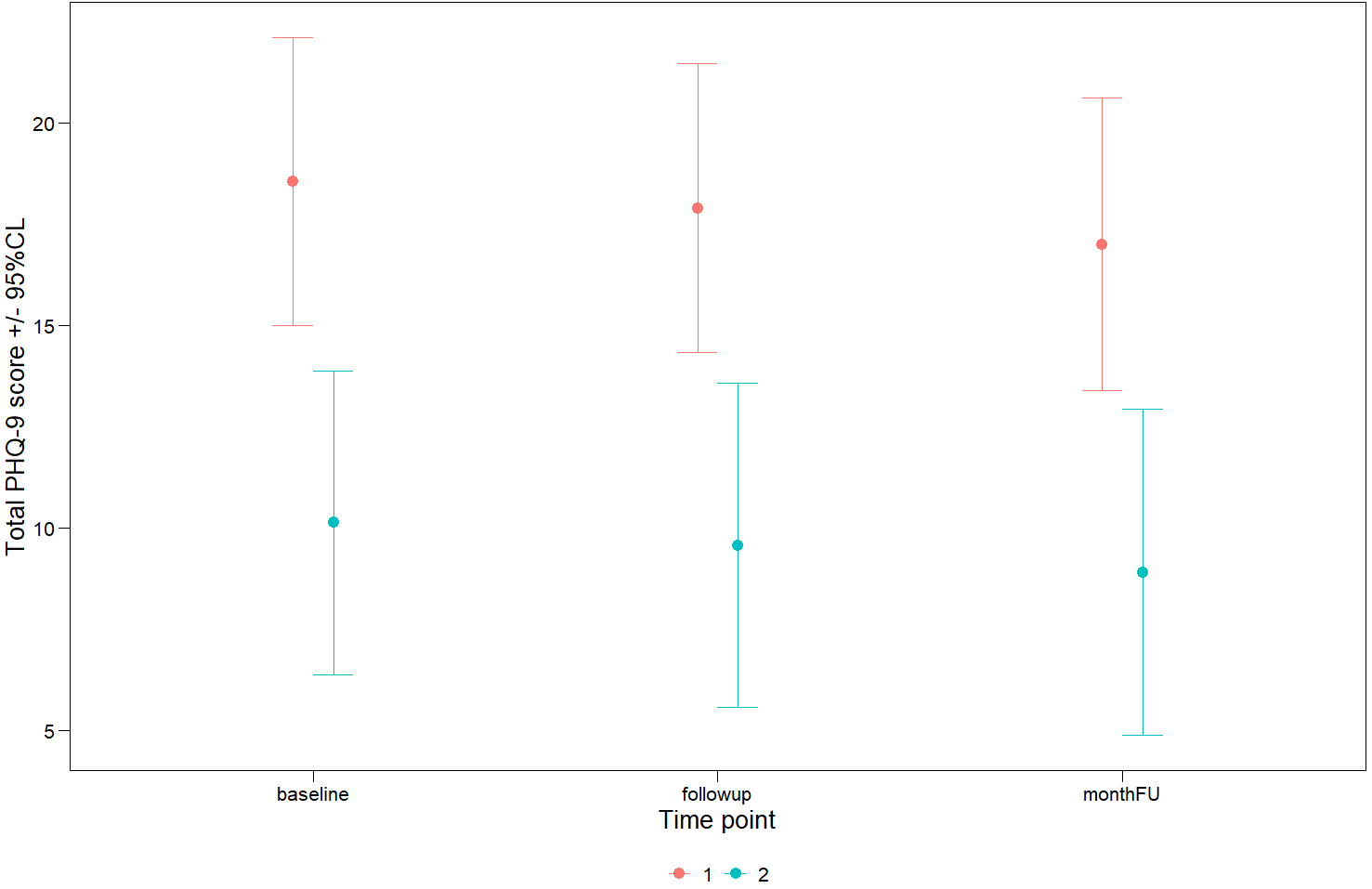


**Fig A.** Marginal means for PHQ-9 score plotted by condition and time

For effects of EW on WHODAS scores, after controlling for session number, the effect of condition was statistically significant. As with PHQ-9 scores, it appears that EW participants had less functional impairment than control participants, including at baseline.

**
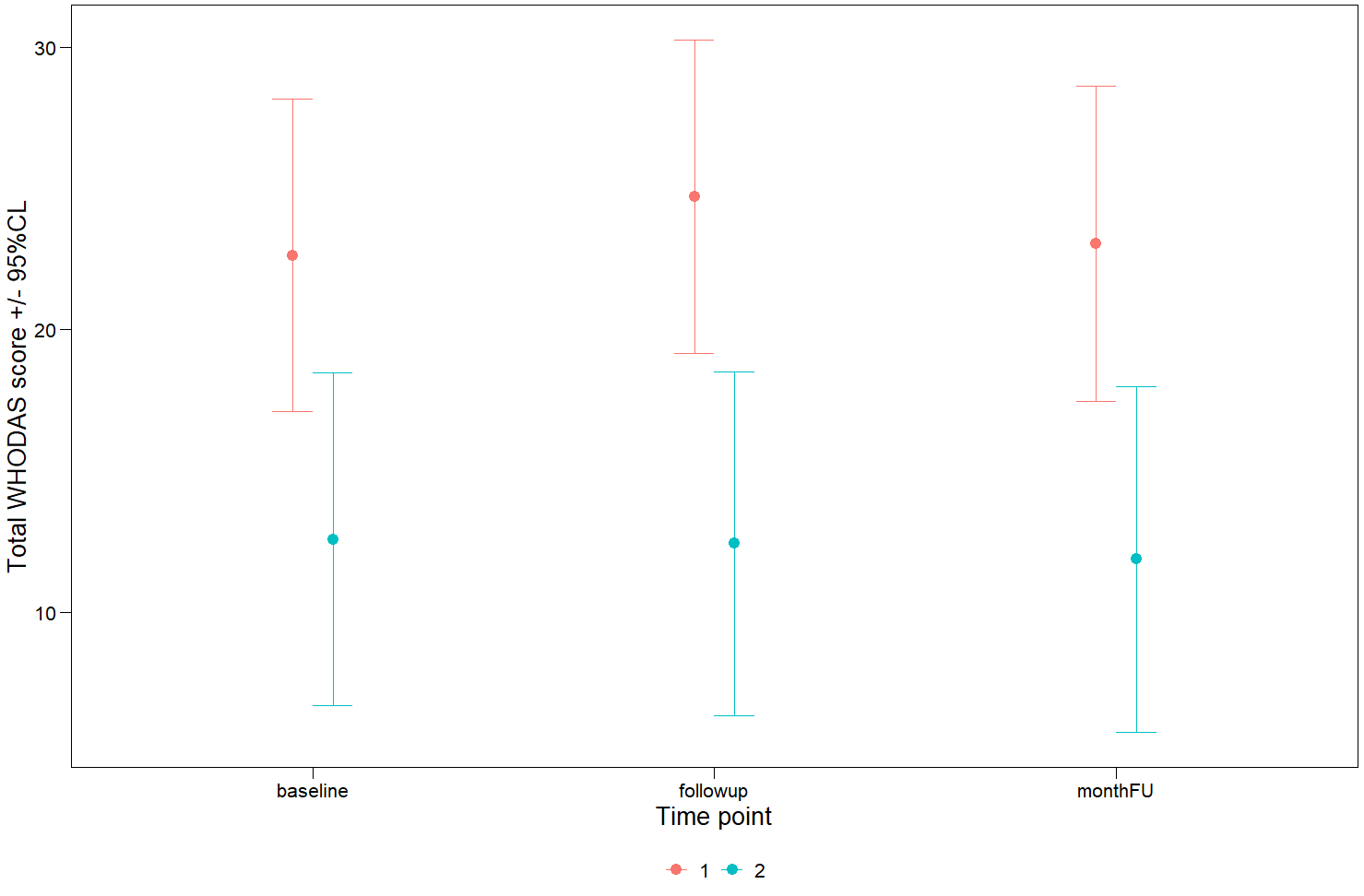
**
 **Fig B.** Marginal means for WHODAS scores plotted by condition and time

For effects of EW on problem complexity, completing more sessions was related to higher problem complexity (*r*=2.27, *SE*=0.62, *p*=.001), and after controlling for this association, the main effect of condition was statistically significant (specifically, problem complexity was lower in the EW condition at each time point, including baseline).


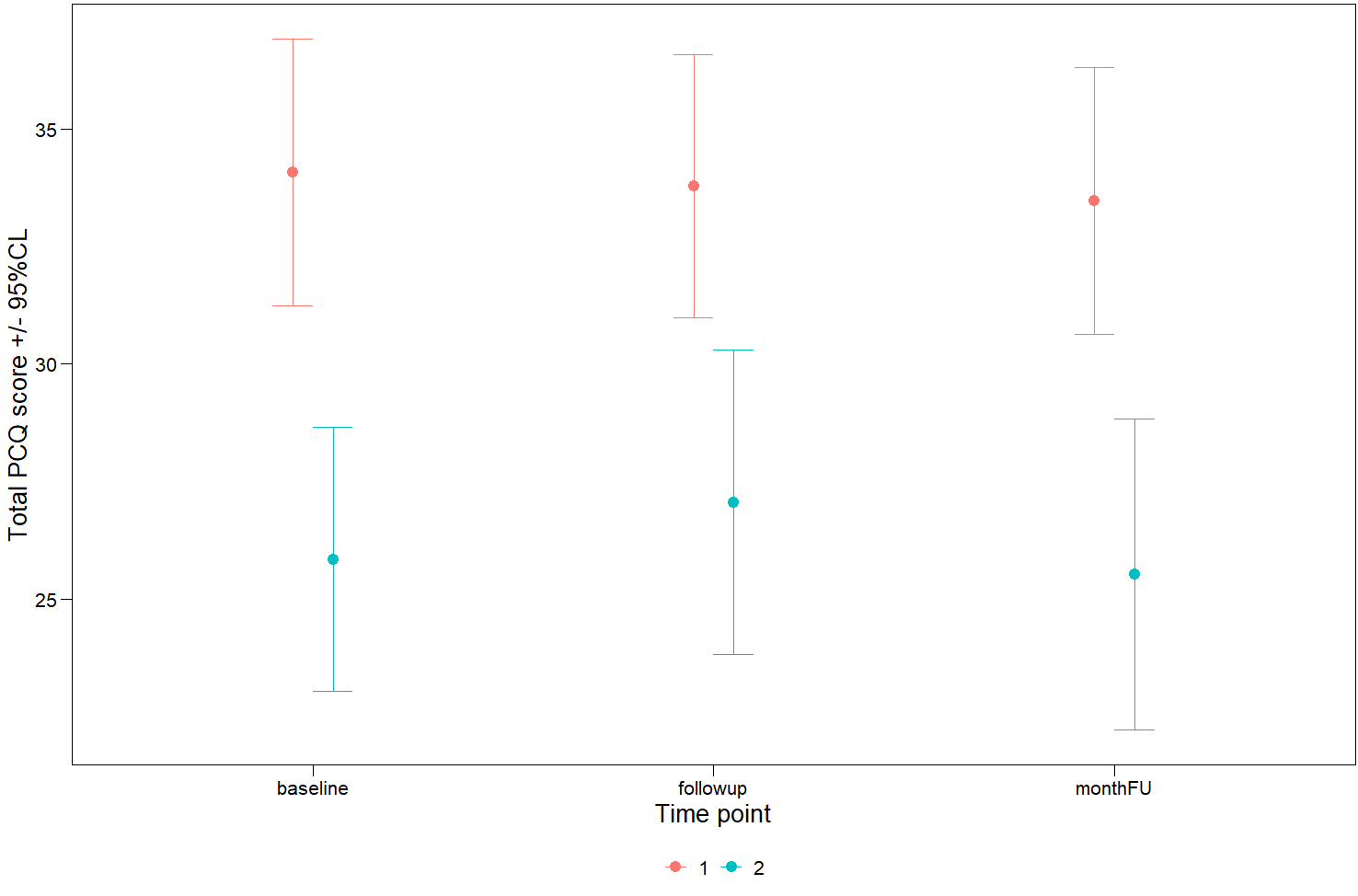


**Fig C.** Marginal means for PCQ scores plotted by condition and time
