# Supplementary material for: Feasibility, efficacy, and perceptions of an online writing intervention in patients with depressive disorders: A randomized, multi-methods pilot study
Source: PLOS Ment Health. 2025 Jul 31;2(7):e0000245. doi: 10.1371/journal.pmen.0000245 (PMC12798339; doi:10.1371/journal.pmen.0000245)

# S6 File

***Depression severity stratified by value group***

Mean PHQ-9 scores for EW participants at each time point, stratified by value group


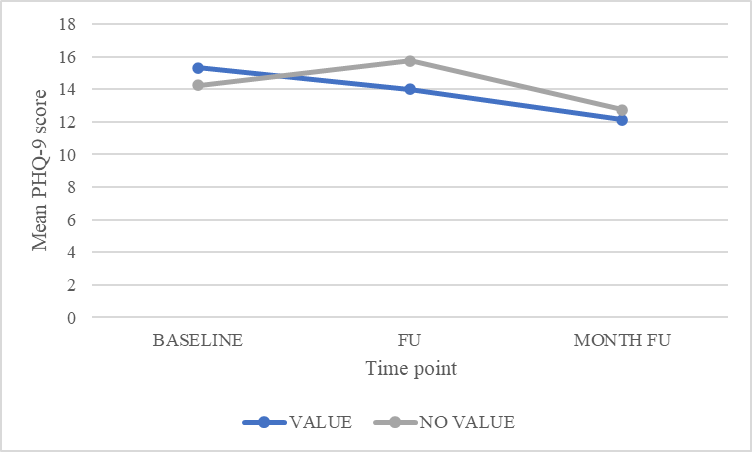

Supplement: S6 File — (DOCX) [file pmen.0000245.s006.docx]
