# Supplementary material for: Feasibility, efficacy, and perceptions of an online writing intervention in patients with depressive disorders: A randomized, multi-methods pilot study
Source: PLOS Ment Health. 2025 Jul 31;2(7):e0000245. doi: 10.1371/journal.pmen.0000245 (PMC12798339; doi:10.1371/journal.pmen.0000245)
Supplement: Attachment — Submitted filename: Response to reviewers_03.24.2025.docx [file pmen.0000245.s008.docx]

Dear Dr. Teles,

Thank you for the opportunity to revise our submission to PLOS Mental Health, entitled “Feasibility, efficacy, and perceptions of an online writing intervention in patients with Major Depressive Disorder: A randomized, multi-methods study” (PMEN-D-24-00481).

We thank the reviewers for their insightful questions and comments, and we provide our responses below.

Best,

Marta Maslej
cc: Abigail Ortiz, Paul Andrews, Benoit Mulsant **Reviewer 1**
 *1. Firstly, although the title [Feasibility, efficacy, and perceptions of an online writing intervention in patients with Major Depressive Disorder: A randomized, multi-methods study] indicates/clarifies that it is a ‘Feasibility’ study only, it is better to add word ‘pilot’ in the title as when the study is ‘pilot’ in nature, many things are ignored [loosely looked at / evaluated; and that more latitude (i.e., leeway, freedom, liberty) in statistical requirements is observed/given] {example: sample size is not a big issue, other methodology issues need not be looked at very rigorously, following of CONSORT guidelines (in case of RCTs) is not strictly observed}.*

We have incorporated this suggestion and revised our title to “Feasibility, efficacy, and perceptions of an online writing intervention in patients with Major Depressive Disorder: A randomized, multi-methods pilot study” (line 2).

*2. I noted that your ABSTRACT is well drafted (in my opinion), but is ‘assay type’. It is preferable [refer to item 1b of CONSORT checklist 2010: Structured summary of trial/study design, methods, results, and conclusions] to divide the ABSTRACT with small sections like ‘Objective(s)’, ‘Methods’, ‘Results’, ‘Conclusions’, etc. which is an accepted practice of most of the good/standard journals [including this one, though the ‘Guidelines to Authors’ might/did not specify an Abstract format, it is desirable]. It will definitely be more informative then, I guess, whatever the article type may be {though Section headings may differ for different Article Types [example: Study Protocol]}.*

We defer to the submission guidelines on this point, which state “while the Abstract is conceptually divided into three sections (Background, Methodology/Principal Findings, and Conclusions/Significance), do not apply these distinct headings to the Abstract within the article file.”

*3. As stated in lines 398-400 that ‘According to a post-hoc power analysis (reported in Supplement S4), our sample size of 23 participants per condition was powered to detect large effects (f=0.47)’ sample size (of 23 participants per condition) will suffice to detect large effects only. Whereas in such studies the ‘effect size’ observed/found generally is very ‘small’ [requiring a large sample size]. According to table-2 on page 158 of Jacob Cohen’s paper “A power primer” in Psychological Bulletin, 1992, vol.:112, pp 155-159 [which is a sort of summary of the excellent book by Cohen himself titled ‘Statistical power analysis for the behavioral sciences’, Academic Press, 1977, New York] even for medium effect size you need n=64 per group (type-I error=0.05, power=80%). Please note that the ‘effect size’ assumed should have some basis (exact reference needs to be quoted) or reasonable/realistic, else the study is very likely ‘not to be able to’ detect a difference despite its presence.* *Very good that this fact (small sample size & consequences) is mentioned at few places in the manuscript [examples: line 553 ‘Although we did not pursue this analysis formally due to our small sample size, mean PHQ-9….’ and lines 618-620 ‘Due to our small sample size, we did not control for these clinical characteristics in our analysis, and they may have impacted outcomes’]. When the outcome is measured in ‘ordinal’ level of measurement then one has to inflate sample size by 10 to 20% and also then the application of suitable non-parametric (or distribution free) test(s) is/are indicated/advisable [even if distribution may be ‘Gaussian’ (also called ‘normal’)]. Though the measures/tools used are appropriate, most of them [examples: Patient Health Questionnaire (PHQ-9), Generalized Anxiety Disorder (GAD-7), WHO-Disability Assessment Schedule (WHODAS), Problem Complexity Questionnaire (PCQ)] are likely to yield data that are in [at the most] ‘ordinal’ level of measurement [and not in ratio level of measurement for sure {as the score two times higher does not indicate presence of that parameter/phenomenon as double (for example, a Visual Analogue Scales VAS score or say ‘depression’ score)}].*

Thank you for pointing us to this interesting paper. We agree with the reviewer that our study is underpowered to detect medium effects, and as noted, we ensure that this point is emphasized in the discussion. The effect size we reported in the Supplement corresponds to a repeated-measures ANOVA, and we have added a reference to this statistic. We used the WebPower package in R to estimate the effect corresponding to the condition by time interaction, based on our current sample size. We have added more detail on this power analysis into the supplement and supported it with references. Regarding the appropriateness of our statistical tests, we examined diagnostics for each model (variance at different levels of fitted/predicted values, normality of residuals, influential datapoints using Cook’s distance), and we did not observe any violations.

*4. The fact brought-out in lines 141-2 {benefits of EW in individuals with MDD may not only reflect changes in symptoms, but they may extend to other outcomes} and in lines 155-7 {it is possible that in studies not reporting statistically significant effects of EW on MDD [19], participants might nevertheless experience benefits related to other aspects of their lives} is appreciated as this is the reality.*

We appreciate this comment; we hope our study encourages researchers to consider other effects of EW on health or wellbeing, such as cognitive and emotional experiences during writing.

**Reviewer 2**
 *1. The introduction often drifts into providing general background information on MDD and the COVID-19 pandemic’s effects on mental health. This inclusion feels somewhat disconnected from the core objective of the study. A clearer articulation of the specific gap the current study aims to fill would make the introduction more focused and compelling.*

We have removed some content elaborating on the effect of COVID-19 on mental health in the introduction, so that the first paragraph is more connected to the specific aims of the study (i.e., to examine the feasibility of administering EW online to address unmet needs for mental health intervention).

*2. The reliance on meta-analyses to report mixed findings of EW’s effects on MDD is important but feels somewhat excessive. The argument for why these mixed results matter in the context of your study is underdeveloped. Rather than only presenting findings, the paper should more directly critique how existing studies' limitations (e.g., homogeneous samples of college students, methodological differences) might affect the applicability of the results to clinical populations.*

We have revised the second paragraph to directly demonstrate how our study addresses a key limitation of the existing literature, which is the lack of study in samples with MDD. We have either removed or moved to the discussion descriptions of methodological differences across studies from the meta-analyses (i.e., number of writing sessions, specificity of instructions, time elapsed between writing sessions; lines 100-109), because these were not directly relevant to our study aims (i.e., we do not examine the impact of these methodological differences on outcomes). We describe various findings supporting the need for conducting studies of EW in samples with severe symptoms or those formally diagnosed with MDD.

*3. The rationale behind the decision not to include a control writing task is not fully convincing. The justification appears to be based on a desire to focus on feasibility, but the lack of a well-defined control condition is a significant methodological weakness.*

Although we cannot change the study procedures or control condition at this stage, we believe our control is appropriate, given we are evaluating whether participants with MDD will complete a writing intervention online, making participants with MDD who are not asked to complete any intervention a relevant comparison group. We found that 6 participants dropped out of the study in the EW condition following the first baseline assessment (vs. 0 control participants), which offers insights into the feasibility of EW (suggesting that some participants with MDD did not wish to complete EW, which may be due to the act of writing, writing about emotional topics, or both). If we had found that EW was feasible and there was evidence of its efficacy, a follow-up study could have examined its efficacy relative to a neutral writing condition to assess the specific impact of writing about emotional topics. However, not using a neutral, control writing condition prevents us from studying the impacts of writing about emotional topics, which we have now added as a limitation: “We also did not employ a neutral, control writing task for this reason, making it unclear whether findings are related to the online writing intervention, the emotional nature of the writing topic, or both.” (lines 665-669).

*4. While the hypotheses presented toward the end of the section are clear, they are somewhat redundant with earlier statements. Repetition of expected outcomes could be avoided by refining the hypotheses and linking them more clearly to the study’s specific aims. There are moments where the language is overly dense or technical, making it challenging to follow the logical flow.*

We revised this section to remove some dense/technical language and redundant content. We have also reorganized this section by linking each hypothesis to its corresponding aim.

*6. While participants were excluded if they had cognitive impairments that might hinder completing the writing task, there is no clear justification for why cognitive impairments, particularly those relevant to MDD (such as memory or concentration difficulties), would not be formally assessed during enrollment. This criterion should have been assessed systematically rather than left to clinician discretion, as cognitive impairments are common in patients with MDD. This omission creates a potential confound in the study sample that could influence the feasibility outcomes.*

We asked clinicians not to refer patients with severe cognitive impairments that would prevent them from being able to complete a writing task, since participants who had very limited literacy or could not write would not be able to participate in the intervention. We now clarify in the methods that this exclusion criteria referred to “severe” cognitive impairment or “very limited literacy” (lines 229-230). As noted in Table 2, the minimum number of words written on any session was 54, suggesting that all participants were capable of writing. It is nevertheless possible that some participants with some severe cognitive impairment or limited literacy may still have been enrolled in the study. This could have impacted our feasibility findings and we now note this as a possible limitation (line 665-667). Of note, we report that, during qualitative interviews, some participants reported cognitive challenges as barriers to completing EW (see lines 460-464). We believe that these findings are relevant to understanding the feasibility of administering EW online to patients with MDD and related cognitive symptoms.

*7. Although the study mentions that clinicians were asked not to refer patients with suicidal thoughts or behaviors, there is no formal assessment of this criterion at enrollment. This is a significant oversight, particularly in a population with MDD, where suicidal ideation is prevalent. The lack of a standardized screening process for this risk factor leaves the study vulnerable to potential ethical and safety concerns, especially when administering the intervention remotely. It is essential that a clearer protocol for identifying and excluding at-risk participants is included.*

As we mentioned in line 232, our study has a protocol for following up with participants who reported suicidal ideation. In brief, participants indicating any suicidal ideation on their baseline PHQ-9 questionnaire answered follow-up questions, which determined the degree of risk. If their responses suggested some risk (e.g., a desire to harm/kill themselves or not take necessary precautions to save their life), the participant’s clinician or if unavailable, a trained study team member followed up with the participant within 24 hours to conduct a suicide risk assessment and if necessary, contract for safety and deploy help. For the ethical reasons mentioned by the reviewer, we use similar procedures in all our intervention studies involving patients with MDD (who are at risk of being suicidal). We have recently reported the likelihood that participants report suicidal ideation that require further clinical attention in a study focused on a patient population similar to our EW study (as reported in Tavakoli et al, 2025, reference provided below). The study’s first author also screened EW text content for evidence of suicide risk, and if this was found, a similar protocol was used to follow up with participants within 24 hours. We have now added details on this protocol into the Supplement (S1), and we refer to this protocol in the revised paper (line 228).

Tavakoli E, Xiang A, Husain MI, Blumberger DM, Kloiber S, Mueller DJ, Ortiz A, Perivolaris A, Mulsant BH. Challenges Related to the Implementation of Measurement-Based Care for the Treatment of Major Depressive Disorder: A Feasibility Study. *Pharmacopsychiatry.* 2025 Feb 25. doi: 10.1055/a-2508-5757. Epub ahead of print. PMID: 39999977.

*8. Excluding participants based on the ability to understand, speak, or write in English should be better justified. In a multicultural context like Canada, where diverse linguistic populations are present, this may inadvertently exclude a significant portion of the population who could benefit from the intervention.*

We have added the following justification for excluding participants who could not understand, speak or write in English: “We did not have access to translation services, so these criteria ensured participants could provide informed consent and complete questionnaires and qualitative interviews. It also enabled us to screen EW texts for evidence of suicide risk” (lines 225-228).

*9. The justification for using postal codes should be more detailed, and it would be helpful to discuss potential limitations of this approach.*

Area-level income measures have been used as proxies of socioeconomic status in studies of mental health, including in the province of Ontario where our study was completed (e.g., see Kurdyak, et al. Incidence of access to ambulatory mental health care prior to a psychiatric emergency department visit among adults in Ontario, 2010-2018. JAMA Network Open, 2021; 4(4), e215902-e215902).

We have revised the paper to highlight that this is a measure of neighbourhood income (line 242). We acknowledge that area-level income measures do not always map on to individual-level measures, and we now mention this in “Limitations” (lines 656-658). *10. The PCQ rationale and validity need further clarification. Furthermore, the use of a single item per domain (e.g., “These problems have left me in a dilemma”) may oversimplify complex personal issues, and a justification for the use of this approach, as opposed to a more comprehensive measure, is needed.*

We describe our rationale for using the Problem Complexity Questionnaire - i.e., its use in Canadian samples and to measure perceived complexity of personal problems in samples with MDD; see lines 270-271. Its psychometric properties have been examined in an MDD sample and it had acceptable mean inter-item correlation and excellent internal consistency, as reported by Sevcikova and colleagues (2020) (this publication is referenced in our manuscript). Although we cannot change our study measures at this time, we have added information on the psychometric properties of this scale and a reference to the relevant publication (lines 271-273).

*11. The control group, which did not complete any writing task, is critical to assess the efficacy of the writing intervention. However, the justification for this control condition is weak. The section mentions that the aim was to focus on feasibility, but this raises the question of whether omitting a proper control group could confound the study's ability to draw valid conclusions about the efficacy of the intervention.*

We have added description of how our choice of control limits interpretation of our conclusions related to efficacy in the Limitations section (lines 667-669). However, we do not believe this choice prevents us from drawing valid conclusions about the efficacy of the intervention – our findings suggest that in our sample of participants, EW did not decrease symptoms of depression/anxiety, functional impairment, and perceived problem complexity, as compared to completing no writing task/intervention. We agree that if we had found that EW was associated with improvement, it would have been unclear whether this improvement was related to the emotional nature of the writing task. As per above, this would have needed to be examined in a subsequent study.

*12. The instructions for the writing task are somewhat vague in places, particularly in terms of guiding participants on how to reflect on and process their emotions. The phrase "write your very deepest thoughts and feelings" is potentially overwhelming for individuals with MDD, as it may prompt emotional flooding rather than therapeutic processing. There is no discussion of how the task was adapted for the target population or how it might be modified for individuals who experience higher distress or difficulty engaging. Further elaboration on how the writing prompt was designed to be both therapeutic and manageable is needed.*

We did not design the writing prompt; we used a standard version of the EW instructions, which were adapted from seminal EW research (Pennebaker & Beall, 1986). These instructions were also employed in the single previous study of EW in samples with MDD, which found a decrease in symptoms related to the intervention (Krpan and colleagues, 2013). This is mentioned in lines 292-293, and these publications are referenced in the manuscript.

*13. The follow-up procedures appear to be standard, but the criteria for determining dropout versus non-completion are underexplained. Participants who did not respond after two reminders were assumed to have dropped out, but what was done with those who responded late or partially completed the intervention is unclear. There is no discussion of how partial participation was handled in the analysis, which could impact the interpretation of feasibility and efficacy. There is no clear explanation of how missing data will be handled, especially in the case of dropouts or partial responses. Given the challenges with participant engagement in online interventions.*

Participants who responded to reminders or completed the questionnaires or intervention late (i.e., within any period of time) were included (described in lines 314-316), which also accounts for the variation in adherence to the EW protocol. Missing data was handled with maximum likelihood estimation (lines 338-339) (as described in our response to query 15 below).

*14. What is the rationale for selecting these models over simpler alternatives (e.g., repeated measures ANOVA)? A brief discussion on why mixed effect models are the most suitable choice for the data structure.*

We chose to use mixed effect models with maximum likelihood estimation so that participants with some missing questionnaire data could still be included in the analysis, and because the two groups did not have an equal number of participants (due to more participants dropping out from the EW condition). Repeated measures ANOVAs would require us to perform listwise data deletion and requires a balanced number of participants per group.

*15. In the per-protocol analysis, there is a focus on the number of completed sessions and text length as predictors, but there is no mention of how these variables will be interpreted in the context of clinical significance. While statistical significance is important, it would be useful to link these measures to real-world improvements in patient outcomes.*

We include these variables to account for the variations in number of completed EW sessions and engagement with the EW task (using text length as a proxy). We believe the related findings could provide insight into whether more online EW sessions predicted better outcomes, as was found in prior research (Reinhold et al., 2016; Guo et al., 2023, both referenced in the manuscript). These findings could also point to text length as a potential predictor of EW efficacy, given that more content written could signal increased engagement or exploration of emotions and thoughts. However, we did not find these factors to be statistically significant predictors, suggesting they would not lead to any clinically relevant effects in this small sample. Thus, we do not discuss these measures in the context of real-world improvements in outcomes.

*16. The qualitative data section could benefit from more detail on how the analysis will be conducted.*

We expanded on our description of each step of the qualitative analysis process (lines 335-365).

*17. The sample size (63 participants) is relatively small, and the significant attrition (from 63 to 46 participants) needs more in-depth discussion. Why did nearly 27% of participants drop out?
Are the results generalizable given the significant loss to follow-up?*

As shown in Figure 1, most participants dropped out after agreeing to participate but before completing the baseline questionnaires (5 in the EW group, 4 in the control group), and an additional 6 participants in the EW condition dropped out during the intervention. These attrition rates are comparable to other studies of EW conducted online (as we describe in lines 532-533). For instance, in one study of postpartum women (Ayers et al., 2018, referenced in our manuscript), attrition rates prior to the completion of baseline questionnaires have been as high as 50%, with attrition rates in online EW conditions being over 25%. As we stopped contacting participants after two reach attempts, we do not know why they dropped out (although we were able to obtain a reason from one participant assigned to EW, who believed it would be detrimental to their wellbeing - see lines 403-404).

*18. The use of abbreviations like "EW" and the inconsistent application of full terms and abbreviations may confuse readers.*

We have reviewed the paper to ensure the use of full terms the first time an abbreviation is mentioned, and for the consistent use of abbreviations. We refer to the term expressive writing quite a few times in the paper (~250 times), so we feel it is appropriate to abbreviate this term, and it is typically abbreviated as EW. We also use common abbreviations for disorders (MDD, GAD) and outcome measures (PHQ-9, GAD-7, WHODAS, PCQ). We have removed abbreviations for any terms only referred to once (i.e., CBT).

*19. There is some redundancy in the reporting of demographic characteristics.*

We revised our reporting of demographic characteristics to remove redundancies (lines 388-390).

*20. The authors fail to discuss the implications of the statistical results in sufficient depth. Are there alternative explanations, such as the low engagement with the EW task or methodological flaws, that could account for these results? Did the low engagement levels contribute to the null findings?*

We agree with the reviewer that findings with respect to efficacy may be related to various methodological reasons, which we have described in the discussion:
- participant non-adherence to the EW protocol (first paragraph, line 522), including not writing for the required 20 minutes (lines 535-536 and line 567), or not writing on consecutive days (line 569-571)
- the online method of administration (vs a prior study finding a large effect of in-person EW in decreasing depressive symptoms in a sample with MDD) (line 554-562)
- our sample being underpowered to detect small or medium effects of online EW in this sample (line 562-564)
We have also incorporated the reviewer’s point that many participants may not have found the task to be engaging, which may have limited its efficacy (lines 578-579).

*21. Clarify how the power analysis was conducted and why the sample size of 23 participants per condition was considered adequate?*

As indicated explicitly in the text, we conducted our power analysis after completing the study (i.e., post-hoc) to help readers interpret our findings. One prior study of EW in a sample formally diagnosed with MDD (Krpan et al., 2013, referenced in the manuscript) found large effects of EW both immediately after writing and at follow up (e.g., *np^2^* = 0.17 and *np^2^* = 0.12 respectively). Our sample of *N*=46 participants had 80% power to detect an effect size of this magnitude. However, this was a study of in-person EW and it is possible that online EW may be associated with smaller effects. As discussed above, we wanted to ensure that readers knew our study was underpowered to detect small or medium effects (as noted in lines 430-432 and again discussed in lines 563-564).

*22. Why was there such variation in completion rates and writing duration? How can the authors account for the skewed distribution of session completion, with many participants failing to write for at least 20 minutes per session?*

Participants were instructed to write for 20 minutes and on consecutive days, but we could not enforce these rules due to administering the intervention online and the voluntary nature of the research. This resulted in variation in completion rates and writing durations. We included participants in our analysis who were not adherent to the protocol due to our focus on feasibility; under realistic conditions, participants may not adhere to instructions related to the timing or length of EW. We attempt to control for these variations in our pre-protocol analyses, by accounting for the number of sessions and word count in the effect of EW on outcomes (see above). However, we do not control for writing durations, as participants were free to pause writing sessions and return to them later, which resulted in some very long writing durations (explained in lines 410-411).

*23. What changes could be made to make EW more effective, and how might these suggestions influence the design of future trials? The authors provide useful suggestions for improving the EW protocol, including offering more specific instructions and integrating clinical oversight. However, these suggestions seem to assume that the EW protocol can be improved by simply making it more structured or personalized, but it’s not clear if this would address the underlying limitations of the intervention.*

We have provided information on potential changes that could be made to EW, based on feedback gathered in qualitative interviews with EW participants (described in lines 501-514), and a discussion of this feedback (lines 620-645). We do not assume that this would make EW efficacious: future studies would be needed to assess whether these changes would lead to increased engagement or adherence to the protocol, and in turn larger decrease in symptoms. The need for additional studies apply to any interventions that is being modified.

*25. The writing text feels overly dense and complex, particularly when discussing findings from previous studies and the nuances of participant feedback.*

We have revised the discussion in an effort to make the writing simpler and more concise.
