# Supplementary material for: Feasibility, efficacy, and perceptions of an online writing intervention in patients with depressive disorders: A randomized, multi-methods pilot study
Source: PLOS Ment Health. 2025 Jul 31;2(7):e0000245. doi: 10.1371/journal.pmen.0000245 (PMC12798339; doi:10.1371/journal.pmen.0000245)
Supplement: Attachment — Submitted filename: Marta Maslej - Response to Rev2 - May 23, 2025.docx [file pmen.0000245.s009.docx]

Dear Dr. Teles,

Thank you for the opportunity to revise our submission to PLOS Mental Health, entitled “Feasibility, efficacy, and perceptions of an online writing intervention in patients with Major Depressive Disorder: A randomized, multi-methods study” (PMEN-D-24-00481).

We are grateful to the additional reviewer for their new questions and comments, and we provide our responses below.

Best,

Marta Maslej
cc: Abigail Ortiz, Paul Andrews, Benoit Mulsant **Reviewer 3**

- *A major question I have about this study is in relation to the focus and inclusion criteria. It states in the title that the focus is patients with MDD, and this is included as an inclusion criterion (i.e., formal diagnosis of MDD from a psychiatrist), but unless I am mistaken only 71% of participants have a diagnosis of MDD with a further 24% having a diagnosis Persistent depressive disorder/Dysthymia. Therefore, the inclusion criteria, title and other parts of this manuscript do not reflect the clinical characteristics of the participants. Please can you clarify how participants without a MDD diagnosis were recruited if this was part of the inclusion criteria (i.e., a diagnosis of MDD from a psychiatrist)? Given that only 71% of the participants had a diagnosis of MDD, I would consider removing any mentions that the findings presented in this manuscript are specific to MDD only to perhaps "mental health outpatients"? It appears that only 48 of the participants who could have their diagnoses retrived from the EHR (i.e., 51) had a recorded diagnosis of a depressive disorder (e.g., either MDD or Persistent depressive disorder/Dysthymia) and instead their primary disorder may be non-mood related. In this case it may also be worthwhile to add to your manscript literature examining the benefits of EW for disorders other than depressive disorders as your study appears to also examine due to the clinical characteristics of the sample.*

Due to this reviewer’s concern, two of the authors (MMM and BHM) have rechecked the diagnoses of the participants, and have reached a consensus on the diagnoses of all the participants for which it was not clear (e.g., because different diagnoses were documented in different parts of the chart). We have now clarified in the Methods the following:

“Psychiatrists or psychotherapists were asked to refer patients who have been diagnosed with MDD” (lines 193-194) … “In this pilot feasibility study, we also did not formally assess patients for a diagnosis of Depressive Disorder with a structured, clinical interview” (lines 209-210)

We have also revised the ‘Descriptive’ section of our Results to reflect the updated clinical information based on our review:

“Diagnosis and treatment information extracted from health records for 53 participants who completed the baseline questionnaire are provided in Supplement S3 (one participant from the initial sample of 54 participants did not consent to our review of their health records). Of the 53 remaining participants, 41 (77%) participants had a diagnosis of MDD, 10 (19%) had a diagnosis of PDD, and one was given a diagnosis (by a psychiatrist) of “depression” with a PHQ-9 score of 24; this latter participant was retained in our analysis with a diagnosis of Depressive Disorder Not Otherwise Specified (NOS). One participant with a diagnosis of GAD did not have a Depressive Disorder and was excluded from the analysis. The participant who did not consent to our review of their health records was retained in the analyses with a diagnosis of Depressive Disorder NOS.” (lines 364-373) … “We could not confirm a diagnosis of Depressive Disorder (MDD or PDD) in one patient who was randomized to the EW intervention” … “This patient was excluded from the analysis, leaving a final sample of 53 participants who completed the baseline questionnaire, and 45 who completed the study.” (lines 353-356)

Because we could confirm a diagnosis of MDD, PDD, or Depressive Disorder NOS for all but one of the 53 retained participants, we have revised the paper to refer to “Depressive Disorders” (vs MDD) in the title and the text. We have updated our analyses to exclude the participant who did not have a Depressive Disorder in the health record, and revised the Results section to update the findings and statistics. In the Discussion (Limitations), we also note that we were able to confirm a diagnosis of depressive disorder in all but one participant:

“We did not formally assess participants for a Depressive Disorder, but we were able to confirm this diagnosis as recorded by psychiatrists in the health records of all but one participant.” (lines 655-657).

With respect to the reviewer’s comment about reviewing the literature examining the benefits of EW for disorders other than depressive disorders, we have not done so, but we have now clarified and elaborated on our description of studies examining the benefits of EW for Depressive Disorders, noting in lines 97-109:

“Two studies examining the impacts of EW on depressive symptoms involved participants who may have had Depressive Disorders, but they did not receive a formal clinical diagnosis: one study involved outpatients undergoing psychotherapy who “self-reported their primary presenting problem”, which included depression (19) and the other study recruited visitors to an informational website about mood disorders (i.e., depression and bipolar disorder) (20). Other studies have involved at-risk samples, such as college students with a history of self-reported depression or trauma (21,22). Only one published study examined the effect of EW in a sample formally diagnosed with a Depressive Disorder, administering the intervention in-person for 20 minutes over three consecutive days in participants diagnosed with MDD experiencing a current major depressive episode based on a structured interview (23).

***Abstract***

- *In the abstract, I would consider providing the number of participants who participated in the interview.*

As recommended, we have added the number of participants who participated in the interviews into the abstract (line 34)

- *You state that 54 participants were included in the study, but it would be helpful to clarify that this is baseline data and that 46 participants completed the study.*

As per above, our analysis now includes 53 participants. They were all included in mixed effect modelling, using maximum estimation likelihood to handle missing data. Since the results in the abstract pertain to these models, we use a N of 53 (and not 46) in the Abstract.

***Introduction***

- *In lines 83 and 88 where you have mentioned meta-analyses by specific authors, you may want to provide the year of publications of these meta-analyses.*

As recommended, we have added publication years, which we agree are useful for evaluating the recency of the reviewed literature from these studies (line 86, line 91)

- *On line 108 you mention that online delivery of EW is potentially unhelpful, but it is not clear in the preceding section whether this delivery modality was explored in any of the studies cited. I would suggest removing the word “online” from this sentence.*

As suggested, we removed ‘online’ from this sentence to avoid confusion (pg. 30, line 118).
To clarify the reviewer’s question on delivery modality, online EW has been explored in only one of the studies cited in the preceding section (Baikie and colleagues, 2012), which was not a clinical sample (participants were recruited from an informational website for mood disorders, suggesting they may be seeking help or at-risk). The writing tasks in the study by Koopman and colleagues (2005) were completed by-hand either in the research lab or remotely (but not online).

***Materials and Methods***

- *If the diagnosis of MDD was retrieved from the EHR was it possible to confirm that this was a current diagnosis and not a historic one, as this may have implications for the description of the participants. Additionally, please can you confirm that prior to beginnning CBT with a psychotherapist that all of the participants recruited through this pathway had also been assessed by a psychiatrist as per the inclusion criteria? And if so, was any data available on the time elapsed between the diagnosis from the psychiatrist and enrollment in the study as their symptoms may have improved in this time.*

See response above: clinicians were asked to refer patients with a current diagnosis of MDD. Because the diagnoses in the EHR were documented at various times, we do not believe it makes sense to provide data on time elapsed between “the diagnosis” and enrollment in the study.

- *Was there any co-design of the EW protocol with the population of interest (e.g., those with MDD or clinicians)? It would be helpful to know this to understand elements of the study such as the frequency of EW chosen*.

The EW protocol was not co-designed with patients or clinicians. We used a common version of the EW protocol (i.e., instructions, writing frequency and duration) from seminal EW work (Pennebaker JW, 1997. Writing about emotional experiences as a therapeutic process. *Psychological Science, 8*(3), 162-166.), which was also used in the single previous study of patients with MDD (Krpan et al, 2013) as we describe in lines 265-267). Co-designing an online version of the intervention with MDD patients would be an interesting direction for future research, which findings from our study could inform (we have added this idea into our discussion, lines 614-615).

- *In the ethics statement and data availability section, do you have an ethics approval number?*

Yes, we have added this number to the manuscript (200/2018, line 250)

- *In the descriptive section of the statistical analysis section, please could you clarify what you mean by recoding the racial ethic categories into a binary variable? What were the two binary groups? I see this is described in table 1 but may be helpful to also state in the methods.*

We mean that participants were grouped into one of two groups (i.e., white or racially marginalized) based on their self-reported racial/ethnic group. As suggested, we have clarified this point in the Methods (lines 304-307). Note we have also changed the term ‘racialized’ to ‘racially marginalized’ to reflect the evolving terminology.

- *As part of feasibility and efficacy, did you collect any data about the reminders (e.g., the rate of response following the reminder 1 or 2)? This may be good to report for future studies which may build off your methods/findings if this is an important tool for encouraging engagement, especially as the reminders were reported to be useful in the qualitative data.*

We did collect these data. As suggested, they have now been added to the Results (lines 391-395) and Table 2.

- *Please confirm how codes were identified in the qualitative data. Was this done by a single member of the research team, or was it multiple under blinded conditions and then compared?*

Codes were identified by a single member of the research team (the first author, MMM), which we now clarify in line 347.

***Results***

- *I wonder if it would be possible to present the engagement data (i.e., number of sessions completed) as a figure? (e.g., a Sankey)? It is a little hard to follow the engagement when it is written.*

The flow of participants through the study (including number of participants completing the four writing sessions) is depicted in Figure 1, which uses the COnsolidated standards of REporting trials (CONSORT) formatting, a standardized diagram for reporting participants in trials. The number (percentage) of participants completing each EW session is also provided in Table 2. We believe that adding a Sankey or related figure would be redundant to the information presented in Figure 1 and Table 2, so we have not made this change.

- *It would be helpful to also add the intervals between sessions of EW to Table 2 if possible.*

We have added this information to Table 2 (median days and range).

- *I would consider updating Table 3 to better reflect the “value” and “no value” groups as you define them. It isn’t very easy to quickly determine how these themes/quotes relate to each group. This would be helpful particularly as you are using these groups to structure the way you have written this section.*

As suggested, we have added content to Table 3 (pg. 24) to clarify that participants classified into the ‘value’ group must have reported effects of EW related to themes “Emotional offloading or release” and/or “Cognitive processing or perspective” (i.e., having found some value in EW), even if they noted effects of EW falling under the other themes (“Emotional re-experiencing” and/or “Cognitive Pitfalls”). Participants in the no-value group only reported effects of EW falling under these two latter themes, and not the themes related to “Emotional offloading or release” and/or “Cognitive processing or perspective”.

***Discussion***

- *Line 498 onwards you hypothesize about why the intervention did not impact MDD/GAD symptoms. Is it possible that the length of the intervention/study was too short for it to have a significant impact, with it only being 4 opportunities to engage in EW offered across 4 days?*

As per above, we used a standard EW protocol (i.e., 20 minutes of writing over 4 days), which was also employed by Krpan and colleagues (2013) in their study of participants with MDD. However, it is possible that the intervention was too short. These points are raised later in the Discussion where potential improvements to the intervention are suggested - the point that more writing sessions may have been useful was mentioned by some participants in qualitative interviews (see lines 622-623). This is congruent with the findings from one meta-analysis that suggest a greater effect of EW on depressive symptoms with more sessions of writing. Relatedly, we discuss how a longer duration may be useful for some individuals (lines 624-626).

- *Is it possible that there are some cognitive symptoms associated with MDD which would make certain individuals inappropriate for EW as an intervention, as is also reflected in your qualitative data? Should this be considered in future research examining the efficacy of EW?*

Indeed, it is possible that EW may be more challenging for individuals with specific cognitive symptoms (e.g., issues with focus, concentration or memory) and it was noted by one participant. Future research may examine whether symptom type moderates an effect of EW, and if so, whether changes can be made to the EW protocol (e.g., offering supports) to accommodate these challenges. We have added this point to the discussion where we discuss potential moderators of the effect of EW (see lines 587-590) and participants who may particularly benefit from further guidance or support (line 632-633).

***Limitations***

- *Why in particular antidepressants? Surely psychotherapy is more likely to impact on EW with this potentially improving the skills potentially required to engage in EW?*

We specified antidepressants as potentially interfering with the effects of EW (vs psychotherapy as facilitating the effects of EW), given we found no effect of EW on depressive symptoms. There have been some suggestions in the literature that antidepressants may interfere with effects of psychotherapy (see Hollon et al 2020; Hollon et al 2021, see below), and if that is true, the fact that half of our sample was taking an antidepressant is a potential limitation of our study.

Hollon, S. D. (2020). Is cognitive therapy enduring or antidepressant medications iatrogenic? Depression as an evolved adaptation. *American Psychologist, 75*(9), 1207.

Hollon SD, Andrews PW, Singla DR, Maslej MM, Mulsant BH (2021). Evolutionary theory and the treatment of depression: It is all about the squids and the sea bass. *Behaviour Research and Therapy, 143*:103849. doi: 10.1016/j.brat.2021.103849. Epub 2021 Mar 27. PMID: 34102409.

***Conclusions***

- *Line 610: I’d consider replacing “in its current state” with “as deployed in the current study” given your limitations.*

We have made the suggested change (line 666) even though we believe that “in its current state” was correct because our EW protocol (i.e., instructions, frequency, duration) aligns with the way it is typically implemented currently – i.e., following (Pennebaker, 1997).
